# Intranasal Treatment of Central Nervous System Dysfunction in Humans

**DOI:** 10.1007/s11095-012-0915-1

**Published:** 2012-11-08

**Authors:** Colin D. Chapman, William H. Frey, Suzanne Craft, Lusine Danielyan, Manfred Hallschmid, Helgi B. Schiöth, Christian Benedict

**Affiliations:** 1 Department of Neuroscience, Uppsala University, Box 593, Husargatan 3, Uppsala, Sweden; 2 Alzheimer’s Research Center at Regions Hospital HealthPartners Research Foundation, St. Paul, Minnesota USA; 3 J. Paul Sticht Center on Aging Dept. of Internal Medicine Section on Gerontology & Geriatric Medicine Wake Forest University School of Medicine, Winston-Salem, North Carolina 27157 USA; 4 Department of Clinical Pharmacology, University Hospital of Tübingen, Tübingen, Germany; 5 Department of Medical Psychology and Behavioral Neurobiology, University of Tübingen, Tübingen, Germany

**Keywords:** central nervous system, insulin, intranasal administration, oxytocin, stem cells

## Abstract

One of the most challenging problems facing modern medicine is how to deliver a given drug to a specific target at the exclusion of other regions. For example, a variety of compounds have beneficial effects within the central nervous system (CNS), but unwanted side effects in the periphery. For such compounds, traditional oral or intravenous drug delivery fails to provide benefit without cost. However, intranasal delivery is emerging as a noninvasive option for delivering drugs to the CNS with minimal peripheral exposure. Additionally, this method facilitates the delivery of large and/or charged therapeutics, which fail to effectively cross the blood-brain barrier (BBB). Thus, for a variety of growth factors, hormones, neuropeptides and therapeutics including insulin, oxytocin, orexin, and even stem cells, intranasal delivery is emerging as an efficient method of administration, and represents a promising therapeutic strategy for the treatment of diseases with CNS involvement, such as obesity, Alzheimer’s disease, Parkinson’s disease, Huntington’s disease, depression, anxiety, autism spectrum disorders, seizures, drug addiction, eating disorders, and stroke.

## DIRECT INTRANASAL DELIVERY TO THE CENTRAL NERVOUS SYSTEM (CNS): TARGETING WITH MINIMAL INVASIVENESS

Traditionally, neurological disorders, like many bodily disorders, have been treated through peripheral administration (predominantly oral administration). However, there are a variety of issues with using peripheral administration to treat CNS diseases. Most significantly, it is difficult to impossible for many molecules, particularly large and/or charged ones, to enter the brain from the bloodstream due to the blood-brain-barrier (BBB), which keeps foreign materials out (1). Additionally, first-pass metabolism can greatly reduce the bioavailability of any drug taken orally, to the point where only a small amount of active drug actually reaches the circulatory system and ultimately the brain (2). Peripherally administered drugs can also take a significant amount of time to reach the brain, so that in acute situations, such as seizures, patients suffer and in some instances face other serious complications—including an increased chance of mortality—while waiting for drug delivery. Plasma protein binding, another consequence of systemic administration, can also affect both the duration and intensity of a drug’s action, reducing its ability to efficiently cross the BBB (3). The final concern with systemic administration is the production of unwanted, peripherally-induced side-effects. Compounds such as insulin, which have a variety of desirable CNS effects, also induce dramatic systemic effects, which can in some cases be problematic (4).

As an alternative, intracerebroventricular injection can deliver drugs directly to the brain; however, it is highly invasive and therefore not realistic for clinical applications (5). On the other hand, intranasal administration, especially to the upper portion of the nasal cavity, has been shown to achieve direct CNS delivery of a variety of compounds without invasiveness or major complications (6–9). In addition, it causes rapid increases in CNS levels of these compounds, and for some—such as insulin—avoids any significant peripheral uptake (10). It may thus represent the most promising, novel, non-invasive method for delivering therapeutic substances directly to the CNS.

## INTRANASAL MECHANISMS

**Fig. 1:**
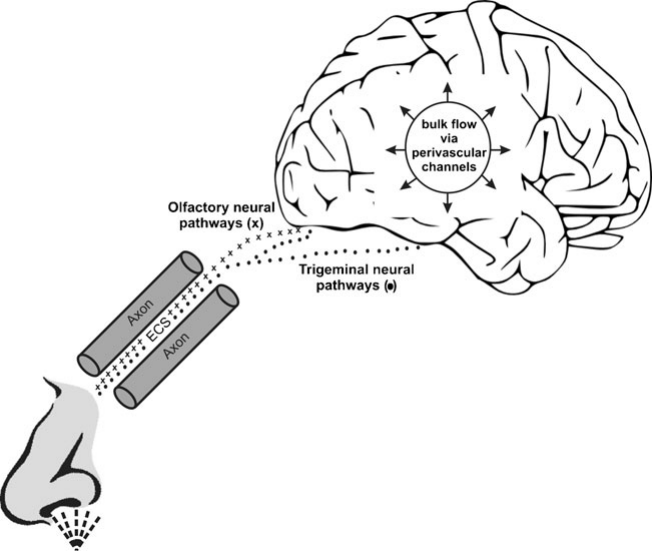
A scheme illustrating the mechanism of nose-to-brain delivery. Unlike the olfactory nerve which terminates in the olfactory bulb, the trigeminal nerve enters the brain through both the pons and the cribriform plate, which allows for drug delivery to both the anterior and posterior regions of the brain (9,12). Transport of substances along the olfactory and trigeminal nerve pathways can happen through both intracellular and extracellular mechanisms (9). However, intracellular transport is a slow process, requiring at best several hours and at worst several days (17,20). Extracellular transport, on the other hand, is rapid and likely accounts for much of the rapid delivery and onset of action observed with intranasal CNS therapeutics (8,21). *Abbreviations*: ECS, extracellular space.

A scheme illustrating the mechanism of nose-to-brain delivery is shown in Figure 1. While the mechanisms involved in intranasal delivery of drugs to the brain are still being elucidated, some of the pathways involved are known. For example, intranasal drugs have been shown to rapidly travel extracellularly along the olfactory nerve pathways leading from the upper part of the nasal cavity directly to the brain (6,9,11,12). This pathway is likely one of the largest contributors to intranasal drug delivery, as drug concentrations in the olfactory bulbs following intranasal delivery are among the highest in the CNS (9,13,14).

The trigeminal nerve pathways are another conduit connecting the nasal passages to the CNS. Unlike the olfactory nerve, the trigeminal nerve enters the brain through both the pons and the cribriform plate, which allows for drug delivery to both the anterior and posterior regions of the brain (9,12). Researchers have demonstrated that a variety of intranasally delivered substances, including insulin-like growth factor 1 (IGF-1), interferon beta-1b and hypocretin-1 (orexin A), result in significant levels of radioactivity in the trigeminal nerve branches, trigeminal ganglion, and the pons, strongly suggesting the involvement of trigeminal nerves in intranasal delivery to the CNS (9,13,15). Additionally, in a recent experiment in mice, the rostral migratory stream has been identified as another potential access path for the CNS delivery of intranasally administered agents (16). However, the relative importance of the various pathways for CNS drug delivery remains unclear.

In general, transport of substances along the olfactory and trigeminal nerve pathways can happen through both intracellular and extracellular mechanisms (9). The intracellular mechanisms include uptake into olfactory sensory neurons (OSNs) within the nasal cavity *via* both diffusion and endocytosis (17,18). OSNs have a demonstrated capacity to endocytose a variety of substances, including some large molecules and viruses (19). However, intracellular transport is a slow process, requiring at best several hours and at worst several days (17,20). Extracellular transport, on the other hand, is rapid and likely accounts for much of the rapid delivery and onset of action observed with intranasal CNS therapeutics (8,21). Within approximately 45–90 min, extracellular transport delivered the tracer peroxidase to the olfactory bulbs (22). Rapid intranasal delivery, as fast as 5–10 min in some cases, of therapeutics to the CNS has been demonstrated with a variety of intranasally delivered drugs, confirming the importance of this extracellular transport mechanism (7,9,10,12,23–25). This rapid speed of transport suggests that for many compounds extracellular convection—along the olfactory and trigeminal nerves—accounts for a significant portion of intranasal delivery to the CNS (9).

In contrast to the intranasal administration of hydrophilic compounds, which typically results in low or no systemic exposure while targeting the brain (10), it can be difficult to avoid systemic exposure with an intranasally delivered small lipophilic molecule. Using a vasoconstrictor formulation may help reduce systemic exposure of lipophilic molecule (26), but studies on the efficacy of this approach are lacking.

## INTRANASAL INSULIN: A MULTI-PURPOSE PHARMACOLOGICAL TOOL TO IMPROVE CENTRAL NERVOUS SYSTEM FUNCTION

Insulin stands as perhaps the most thoroughly investigated compound with regard to intranasal delivery for the improvement of CNS functioning. Intranasally administered insulin appears to benefit a variety of measures, including food intake, body weight, memory, and mood, while avoiding many unwanted peripheral side effects. With regard to memory, initial experiments revealed that 8 weeks of intranasal insulin improves performance on a declarative (delayed recall) memory task (27,28). Further studies have demonstrated that intranasal insulin improves verbal memory in both cognitively impaired older adults and those with early Alzheimer disease (AD) (29–31). In a recently published clinical trial with 104 adults with amnestic mild cognitive impairment or mild to moderate Alzheimer’s disease, 4 months of intranasal insulin administration reduced not only general cognitive decline but also preserved metabolic integrity of the brain, as indicated by cognitive testing and fluorodeoxyglucose positron emission tomography (FDG-PET) (32). These results suggest that targeting the brain insulin pathway by means of intranasal administration of the hormone is a promising therapeutic strategy to improve memory and potentially deter the process of this devastating disease (33–37). Another study demonstrated that intranasal insulin treatment for 1 year appeared to improve the developmental delay in children suffering from 22q13 deletion syndrome that is associated with cognitive impairments, generalized hypotonia and autistic behavior (38). While many of these effects have been demonstrated in both sexes, there is some evidence suggesting that the cognitively enhancing properties of insulin are more pronounced in women than in men (39). Additional evidence suggests that these effects are genotype dependent, such that groups with different genetic risk profiles for cognitive impairment or AD may show different dose-response curves following intranasal insulin administration. For example, memory-impaired adults with the episolon4 allele for Apolipoprotein E (APOE)—a risk factor for AD and impaired cognitive function—show a relative decline in verbal memory following intranasal insulin, in contrast to those without this allele (29). However, the dependency on gender and genotype needs further investigation.

Intranasal insulin has also emerged as a potential treatment for both obesity and diabetes. Eight weeks of daily administration resulted in significant loss of body weight, fat, waist circumference, and leptin levels in men (40). However, this same research failed to find an effect in women, again suggesting that the effects of insulin may be gender-dependent. This conclusion is reinforced by other studies demonstrating the same pattern of results in the acute setting, with men eating less following intranasal insulin administration, while women received a memory boost (39). However, there is also evidence to the contrary. A recent study revealed that intranasal insulin administered postprandially intensified satiety, reduced later intake of calorie dense, palatable foods, and impacted peripheral glucose homeostasis in women, suggesting that intranasal insulin may have some potential in the treatment of obesity and diabetes in both men and women (41). Additional evidence for this assumption comes from its demonstrated ability to increase postprandial thermogenesis and energy expenditure, suggesting that insulin may improve obesity and metabolic syndrome not only through its anorectic properties, but also through thermogenesis (42). Finally, functional magnetic resonance imaging (fMRI) has revealed that intranasal insulin selectively reduces brain activity in memory centers within the brain, such as the hippocampus, in response to food images (43). This could represent another mechanism by which insulin suppresses rewarding food related memories, and as a consequence food intake.

It is emerging that insulin also has stress and mood regulating properties specific to the CNS. For example, a recent study utilizing the Tier Social Stress Test (TSST) found that intranasal insulin attenuates both plasma and saliva cortisol release in response to social challenges, demonstrating its role in regulating the hypothalamic-pituitary adrenal (HPA) axis stress responses (44). Eight weeks of daily intranasal administration also caused significant decreases in participants’ self-rated anger and increases in their self-confidence and general well-being (27). However, there is still a dearth of evidence supporting this connection, and future studies are needed to reveal the particular impact of central nervous insulin on the ongoing regulation of stress reactions and emotions.

## INTRANASAL LEPTIN: AN ANTI-OBESITY AGENT?

Leptin is one of the best known players in the regulation of body weight and appetite (45). Early characterization of this anorectic transmitter lead to the belief that it may serve as a magic bullet solution to obesity. However, systemic administration of leptin has failed to provide any substantial benefits for weight decrease or appetite reduction in obesity (46,47). In addition, peripheral administration of leptin fails to increase leptin concentrations in CSF, suggesting that the problem, at least in part, involves the quantities of leptin able to pass the BBB (48). Based on this reasoning, a handful of recent pre-clinical studies have investigated the possibility that intranasal administration of leptin may enhance its anorectic potential, and the results so far are very positive. In trials using both lean and diet-induced obese (DIO) rats, intranasal leptin has been demonstrated to reduce appetite and induce weight loss (49,50). In addition, these findings were equally substantial in the DIO and lean groups. These experiments also found that this form of administration successfully altered the hypothalamic levels of a variety of regulators of energy homeostasis, including neuropeptide Y, proopiomelanocortin and agouti-related protein. Thus leptin represents one of several compounds with apparent therapeutic potential on the frontiers of intranasal delivery.

## SOCIAL BEHAVIOR AND BODY WEIGHT: THE ROLE OF OXYTOCIN

Oxytocin is another peptide that has been extensively investigated with intranasal administration; however, the areas of this research are constantly expanding. Historically, intranasal oxytocin has been useful for the acceleration and augmentation of contractions during childbirth (51). However, the past decade has revealed a plethora of other potential uses for intranasal oxytocin. Unlike insulin, oxytocin does not present with many unwanted systemic effects; however, because of the size of the molecule it is ineffective to administer it *via* the periphery, as it does not seem to pass the BBB in significant quantities (52). Thus, intranasal delivery provides the most effective method to utilize its various beneficial CNS effects. As vasopressin is known to gain access to the CNS *via* intranasal administration, and oxytocin only differs from vasopressin by two amino acids, it is reasonable to assume that it gains access *via* a similar mechanism (10).

Recent research into oxytocin has largely focused on its role in improving social behavior, and as an extension psychiatric disorders affecting social life. Much of this research was spurred by the seminal work of Kosfeld and coworkers who revealed that oxytocin increases trust in humans, thereby demonstrating its pro-social potential (53). In healthy volunteers, intranasal oxytocin has also been shown to improve performance on the Reading the Mind in the Eyes (RMET) test, which involves detecting social cues from the eye region (54). Follow-up research demonstrated that this effect translates to children and adolescents (aged 12 to 19) with autism spectrum disorders, demonstrating a clear application of oxytocin’s pro-social effects (55). Other research has discovered that intranasal oxytocin improves positive evaluations of appearance and speech performance during exposure therapy for social anxiety disorder (SAD) (56). However, this same study found no significant difference in treatment effects on SAD symptoms and dysfunctional thoughts between oxytocin and placebo, so that further research is needed to demonstrate if different dosing regiments, or use in conjunction with alternative interventions might improve oxytocin’s efficacy in SAD. Another study demonstrated that even the highly intractable borderline personality disorder (BPD) benefits from oxytocin—intranasal delivery attenuated BPD stress reactivity in response to the TSST (57). Perhaps most surprisingly, central oxytocin also improves schizophrenia-induced social deficits (58), including performance on the Brüne Theory of Mind Picture Stories Task (59) and the Trustworthiness Task (60).

While oxytocin’s role in social behaviors is well established, it may have additional therapeutic applications that have yet to be fully explored. In rats, centrally administered oxytocin has been shown to reduce food intake and body weight (61,62). Recent follow up research demonstrated that this effect is particularly pronounced in diet-induced obese rats (63). These results suggest that centrally administered oxytocin may facilitate weight loss in humans, particularly in those with metabolic syndrome, and clinical trials are currently investigating this question (clinicaltrials.gov). There are additional reasons to suggest that oxytocin may be particularly effective in combating obesity associated with Prader-Willi syndrome (PWS). PWS is a congenital disease that produces a variety of undesirable effects, including gross body weight gain (64). Interestingly, PWS is also characterized by dramatically reduced levels of oxytocin in the paraventricular nucleus of the hypothalamus—a region critically involved in body weight homeostasis (65). Thus, central oxytocin may improve this aspect of PWS.

## INTRANASAL OREXIN-A AS A THERAPEUTIC OPTION TO TREAT NARCOLEPSY

In 2004, intranasal hypocretin-1 (orexin A) was first shown to be delivered from the nose to the brain and proposed as a new strategy to treat narcolepsy (16). A study in non-human primates demonstrated that intranasal hypocretin-1 reduces cognitive performance deficits resulting from sleep deprivation (66). However, intranasal hypocretin-1 administration is just starting to be explored in humans. This line of research has focused on narcolepsy—a disorder characterized by impaired or absent CNS hypocretin signaling—and has shown promising results (67). Intranasal hypocretin-1 stabilizes rapid eye movement (REM) sleep and reduces wake to REM transitions in narcoleptics when administered prior to sleep onset (68). Olfactory dysfunction, a well-known aspect of narcolepsy, is also improved by intranasal hypocretin-1 (67). Animal studies have demonstrated that intranasal administration leads to significantly greater tissue-to-blood concentration ratios in all brain regions over 2 h as compared to intravenous (IV) administration. Intranasal administration also increased drug targeting to the brain and spinal cord 5- to 8-fold (69). While these early studies are promising, further research is needed to assess whether the fast acting and potent intranasally delivered hypocretin-1 can aid in the prevention of cataplexy, sleep paralysis, hallucinations, excessive daytime sleepiness, or other symptoms associated with narcolepsy.

## INTRANASAL BENZODIAZEPINES: OPTIMIZING EMERGENCY SEIZURE TREATMENT

Early intervention in a patient who is seizing reduces the chances of both morbidity and mortality (70,71). While IV administration is preferred, most prolonged seizures begin outside of hospital settings. It is thus important for parents and caretakers to have simple, cheap, and effective methods for treating patients experiencing a seizure. Benzodiazepines, such as diazepam, lorazepam, clonazepam and midazolam, are currently the most popular compounds for acutely treating seizures (72,73). IV and intramuscular administration have historically been standard for drug delivery in hospital settings. Outside of the hospital, rectal treatment, which carries with it significant social taboos—which result in delayed treatment and sometimes decisions not to treat—is currently the preferred method of administration (74). However, intranasal benzodiazepines, including midazolam and lorazepam, have been researched extensively as possible replacement treatments, both in and outside of the hospital, and the results have been very promising. For example, intranasal midazolam has been shown to be effective in 87% of patients with prolonged seizures, while rectally delivered diazepam was only effective in 60% (75). More recent work has found that, in 358 pediatric patients randomly assigned one treatment or the other, there was no detectable difference in efficacy between intranasal midazolam and rectal diazepam (76). This same study and others have reported that caregivers found the intranasal treatment significantly easier to use, and it is also noteworthy that intranasal midazolam is markedly cheaper than rectal treatments (77). With these considerations in mind, intranasal delivery appears to be highly effective for treatment of acute seizures, and should be seriously considered as the preferred method of administration both in and outside of hospital settings.

## INTRANASAL NALOXONE: REVERSING OPIOID OVERDOSE AND TREATING BINGE EATING DISORDER

Naloxone has long been the preferred treatment for opioid overdoses; however, parenternal administration brings with it the risk of needlestick injury in a population that is at higher risk for blood-borne viruses (78). Studies have thus been investigating the possibility of intranasal administration in pre-hospital settings. One such study, utilizing 154 patients treated over 1 year by the Central California EMS Agency, found that there was no significant difference in the likelyhood of clinical response between intranasal (66% response) and intravenous (56% response) administration of naloxone (79). While this same study found that the mean time between administration and clinical response was shorter for IV *versus* intranasal delivery (8.1 *vs*. 12.9 min), there was no significant difference in the average time from patient contact to clinical response. Intranasal administration thus presents a safe and effective alternative pre-hospital intervention for reversing the effects of opioid overdose (80).

More recently, Lightlake Therapeutics has conducted a 6-month phase II placebo-controlled trial of intranasal naloxone for the treatment of binge eating disorder with 127 subjects. Patients who received intranasal naloxone had a highly significant reduction in the time they spent binge eating compared to placebo. Additionally, they achieved reduced body-mass indices (BMIs) during the second half of the 24-week trial, and had better perceptions of their binge eating as measured by the Binge Eating Scale (BES) (81). Intranasal naloxone thus appears to provide benefit in both situations involving drug overdose and binge eating.

## INTRANASAL STEM CELLS AS THERAPEUTIC OPTION FOR PARKINSON’S DISEASE, ALZHEIMER’S DISEASE, HUNTINGTON’S DISEASE, STROKE, AND MORE

Perhaps the most exciting recent development regarding intranasal treatment options for CNS disorders is the discovery that intranasal stem cells rapidly reach the CNS and produce therapeutic benefit in animal models. Stem cells have been considered for use in the treatment of a plethora of neurological conditions, including Parkinson’s disease (PD), AD, Huntington’s disease (HD), and stroke (82–85). However, the BBB impairs the ability of stem cells to reach the CNS from the periphery, and surgery is highly invasive and can cause a local inflammatory response that damages the implanted stem cells, leaving noninvasive intranasal administration as one of the most attractive therapeutic options. Preclinical trials have already produced surprising and promising results: the first published experiments demonstrated that intranasally administered bone marrow-derived mesenchymal stem cells (MSCs) reached the brain and cerebrospinal fluid *via* rapid extracellular delivery along the olfactory neural pathway, and this delivery was significantly enhanced by pretreatment of the nasal mucosa with hyaluronidase (86). Following this work, efficacy of this intranasal stem cell delivery and treatment method was demonstrated in three different animal models by three different groups of researchers.

Using a mouse model, researchers demonstrated that 28 days following cerebral hypoxia-ischemia, neonatal animals treated with intranasal MSCs had significantly improved sensorimotor function in the cylinder rearing test (87). MSCs also decreased gray and white matter loss by 34 and 37%, respectively. A second group using a unilateral 6-hydroxydopamine (6-OHDA) lesion rat model of Parkinson’s disease demonstrated that intranasally administered MSCs resulted in the appearance of cells in the olfactory bulb, cortex, hippocampus, striatum, cerebellum, brainstem, and spinal cord with preferential targeting of the MSCs to the lesioned side and damaged areas of the brain (88). Out of 1 × 10^6^ MSCs applied intranasally, 24% survived for at least 4.5 months in the brains of 6-OHDA rats, and 3% of applied MSCs were proliferative 4.5 months after application. Intranasal stem cell treatment increased tyrosine hydroxylase and prevented dopamine loss in the lesioned striatum and substantia nigra and completely eliminated the 6-OHDA-induced increase in tunnel staining in these brain regions. It also decreased the concentrations of multiple proinflammatory cytokines in the lesioned side to levels seen in the intact unleasioned side. Significant and substantial improvement in motor function was also observed following intranasal treatment with the MSCs. Thus, intranasal administration of therapeutic stem cells provides a promising noninvasive alternative to traumatic surgical transplantation and allows for targeted delivery of stem cells to the CNS with the option of chronic treatment (88).

Another research group used a similar rodent model of Parkinson’s disease but a different intranasal delivery method and different method (near-infrared live imaging) for detecting cells in the brain (89). Additionally, they utilized human, as opposed to rat stem cells, in contrast to (88). Although they were able to detect a strong near-infrared signal in the nasal cavity immediately, they lost the signal within 1 h. However, this loss of signal could be explained by a variety of factors, including the fact that human stem cells may have different migratory capacities and paths, and may suffer from an immune response in rodents. It also cannot be excluded that near-infrared live imaging is not sensitive enough to track cell migration into the brain after intranasal or systemic administration, since these types of delivery imply the process of cell migration which in turn means the appearance of single cells distributed within certain areas in the CNS. Two reports failed to show fluorescently labeled cells (enhanced green fluorescent protein (EGFP) or Hoechst 33258) after intranasal delivery of cells in rats and mice (90,91), while 4 other studies did show successful detection of cells using Hoechst 33342 (86,92), EGFP (88) or PKH-26 labeling (a lipophilic dye that stably integrates into the cell membrane, without disturbing its surface marker expression) (87). Thus, the delivery efficacy after intranasal administration should be proven either by demonstration of CNS therapeutic effects or by various detection methods such as detection of radiolabelled stem cells in the brain or DNA analyses of genes (such as green fluorescent protein, GFP) specifically expressed in the stem cells after they reach the brain (88).

Preclinical research has also revealed that MSCs have therapeutic potential in brains damaged by stroke. Hypoxia-preconditioned bone marrow mesenchymal stem cells (HP-BMSCs) delivered intranasally to mice 1 day after an ischemic stroke migrated to ischemic regions as early as 1 h post delivery (91). Within 4 days, BMSCs also reduced the infarct volume and attenuated stroke-related neurological deficits. Additionally, 2 weeks following delivery intranasal BMSCs significantly improved blood flow to ischemic regions. While it has been speculated that intranasal stem cells may improve other conditions that benefit from stem cell treatment, many preclinical and clinical trials are still needed to demonstrate the safety and efficacy of this approach.

## CLINICAL SAFETY OF INTRANASAL ADMINISTRATION

While there are still open questions regarding the mechanics of intranasal administration, it is becoming increasingly clear that this delivery route is safe and effective. A meta-analysis of the safety, side-effects and subjective reactions to intranasal oxytocin revealed that it produces no reliable side-effects, and is not associated with adverse outcomes when delivered in doses of 18–40 IU for short term use (93). Similar reviews and meta-analyses have been published confirming the safety of a variety of intranasal compounds, including steroids, insulin, and midazolam (94–96). As demonstrated in the above meta-analyses, intranasal administration also has a favorable side-effect profile. For instance, although an intravenous infusion of insulin yields to increased brain insulin levels (97), it has also been linked to elevated blood pressure (98), and enhanced hypothalamo–pituitary–adrenal secretory activity (99). In contrast, enhanced brain insulin for shorter time periods signaling by intranasal administration of the hormone has been associated with no changes in blood pressure (100) and dampened hypothalamo–pituitary–adrenal (HPA) secretory activity (27). This example indicates that the intranasal administration method produces less undesirable consequences while still achieving desirable CNS results. However, while intranasal delivery may, in general, produce a favorable side-effect profile as compared to other delivery routes, each drug must be examined for its particular effects on the nasal mucosa, the sense of smell and the immune system as the drug will likely enter not only the CNS but also the nasal associated lymphatics and deep cervical lymph nodes.

## CONCLUDING REMARKS

The benefits of intranasally delivered compounds continue to be discovered. With over 100 clinical trials in the United States alone currently investigating intranasal administration, it is a rapidly growing method of administration (clinicaltrials.gov). The greatest promise appears to lie in compounds such as intranasal insulin and intranasal oxytocin, which are dominating the current list of clinical trials. However, other therapeutics that are still in pre-clinical trials, such as stem cells, also have significant therapeutic potential. The future for intranasal delivery is thus bright, as it represents the most cutting edge way for drugs to be quickly, easily, and non-invasively delivered directly to the CNS.
